# Isoxazole compound ML327 blocks MYC expression and tumor formation in neuroblastoma

**DOI:** 10.18632/oncotarget.19406

**Published:** 2017-07-20

**Authors:** Eric J. Rellinger, Chandrasekhar Padmanabhan, Jingbo Qiao, Brian T. Craig, Hanbing An, Jing Zhu, Hernán Correa, Alex G. Waterson, Craig W. Lindsley, R. Daniel Beauchamp, Dai H. Chung

**Affiliations:** ^1^ Section of Surgical Sciences, Department of Surgery, at Vanderbilt University Medical Center, TN 37232, Nashville, USA; ^2^ Department of Pathology, at Vanderbilt University Medical Center, TN 37232, Nashville, USA; ^3^ Department of Pharmacology and Vanderbilt Institute of Chemical Biology, at Vanderbilt University Medical Center, TN 37232, Nashville, USA; ^4^ Department of Cancer Biology, at Vanderbilt University Medical Center, TN 37232, Nashville, USA; ^5^ Department of Cell and Developmental Biology, at Vanderbilt University Medical Center, TN 37232, Nashville, USA; ^6^ Department of Pediatric Surgery, at Vanderbilt University Medical Center, TN 37232, Nashville, USA

**Keywords:** ML327, neuroblastoma, MYCN, neural crest, epithelial-to-mesenchymal transition

## Abstract

Neuroblastomas are the most common extracranial solid tumors in children and arise from the embryonic neural crest. *MYCN*-amplification is a feature of ∼30% of neuroblastoma tumors and portends a poor prognosis. Neural crest precursors undergo epithelial-to-mesenchymal transition (EMT) to gain migratory potential and populate the sympathoadrenal axis. Neuroblastomas are posited to arise due to a blockade of neural crest differentiation. We have recently reported effects of a novel MET inducing compound ML327 (*N*-(3-(2-hydroxynicotinamido) propyl)-5-phenylisoxazole-3-carboxamide) in colon cancer cells. Herein, we hypothesized that forced epithelial differentiation using ML327 would promote neuroblastoma differentiation. In this study, we demonstrate that ML327 in neuroblastoma cells induces a gene signature consistent with both epithelial and neuronal differentiation features with adaptation of an elongated phenotype. These features accompany induction of cell death and G1 cell cycle arrest with blockage of anchorage-independent growth and neurosphere formation. Furthermore, pretreatment with ML327 results in persistent defects in proliferative potential and tumor-initiating capacity, validating the pro-differentiating effects of our compound. Intriguingly, we have identified destabilization of MYC signaling as an early and consistent feature of ML327 treatment that is observed in both *MYCN*-amplified and *MYCN*-single copy neuroblastoma cell lines. Moreover, ML327 blocked *MYCN* mRNA levels and tumor progression in established *MYCN*-amplified xenografts. As such, ML327 may have potential efficacy, alone or in conjunction with existing therapeutic strategies against neuroblastoma. Future identification of the specific intracellular target of ML327 may inform future drug discovery efforts and enhance our understanding of MYC regulation.

## INTRODUCTION

Neuroblastomas are the most common extracranial solid tumors in children, accounting for 10% of cancer-related deaths in children [1]. Children afflicted with neuroblastoma are risk-stratified based upon age at diagnosis, stage of tumor progression, and biologic features including the presence of *MYCN*-amplification [2, 3]. *MYCN*-amplification is present in ∼30% of all neuroblastomas and overexpression of N-MYC is sufficient to drive neuroblastoma-like tumors in animal models, highlighting the critical role of this oncogene in neuroblastoma development and progression [4, 5].

Neuroblastomas arise from neural crest derivatives and may develop anywhere along the sympathoadrenal axis, most commonly in the adrenal medulla [3]. Neural crest precursors develop from the dorsal neural tube, where the cells undergo epithelial-mesenchymal transition (EMT) to gain migratory potential and populate the sympathetic nervous system [3, 6]. This process is characterized by loss of E-cadherin within adherens junctions and destabilization of tight junctions that mediate cell adhesion. During normal sympathoadrenal development, post-migratory neural crest cells express high levels of N-MYC, where it regulates the ventral migration and expansion of neural crest cells [6]. N-MYC protein levels gradually decrease as the neural crest precursors differentiate into sympathetic neurons, suggesting that sympathoadrenal maturation requires low or absent N-MYC expression [7]. As such, aberrantly high N-MYC is thought to contribute to neuroblastoma tumorigenesis at least in part by promoting a persistent mesenchymal phenotype within neuroblastomas.

The EMT changes observed in early neural crest migration parallels those observed in epithelial carcinoma metastasis [8, 9]. Acquisition of a mesenchymal phenotype has been linked to advanced stage disease, chemoresistance, and radiation resistance in epithelial carcinomas, suggesting that reversal of EMT using small molecules may represent a novel anti-cancer strategy [8–10]. As previously reported, we conducted a high throughput chemical probe screen of 83,200 small molecules to identify novel compounds capable of inducing the re-expression of E-cadherin in colon and lung carcinoma cells exhibiting EMT features [11, 12]. Chemical optimization of an effective initial “hit” yielded approximately 400 chemical derivatives, from which we identified ML327 (*N*-(3-(2-hydroxynicotinamido) propyl)-5-phenylisoxazole-3-carboxamide) as a potent inducer of mesenchymal-to-epithelial transition (MET) in epithelial cancers and that also blocks cancer invasiveness [12–14].

In the present study, we hypothesized that treatment with the MET induction agent ML327 would induce differentiation in neural crest-derived neuroblastoma tumor cells. Herein, we demonstrate that ML327 induces morphological, behavioral, and gene signature changes patterns consistent with differentiation. Intriguingly, we also demonstrate that ML327 arrests neuroblastoma cell growth and transcriptionally suppresses MYC expression in neuroblastomas. Furthermore, we demonstrate that ML327 alters the proliferative potential and tumor-initiating capacity of neuroblastoma cells and that ML327 treatment blocks the growth of established neuroblastoma xenografts. Together, these findings suggest that ML327 is a novel chemical probe that blocks MYC expression in neuroblastomas and may represent a lead compound for further development of novel therapeutics for this aggressive pediatric solid tumor.

## RESULTS

### ML327 initiated G1 cell cycle arrest and cell death in neuroblastoma cells

**Figure 1:**
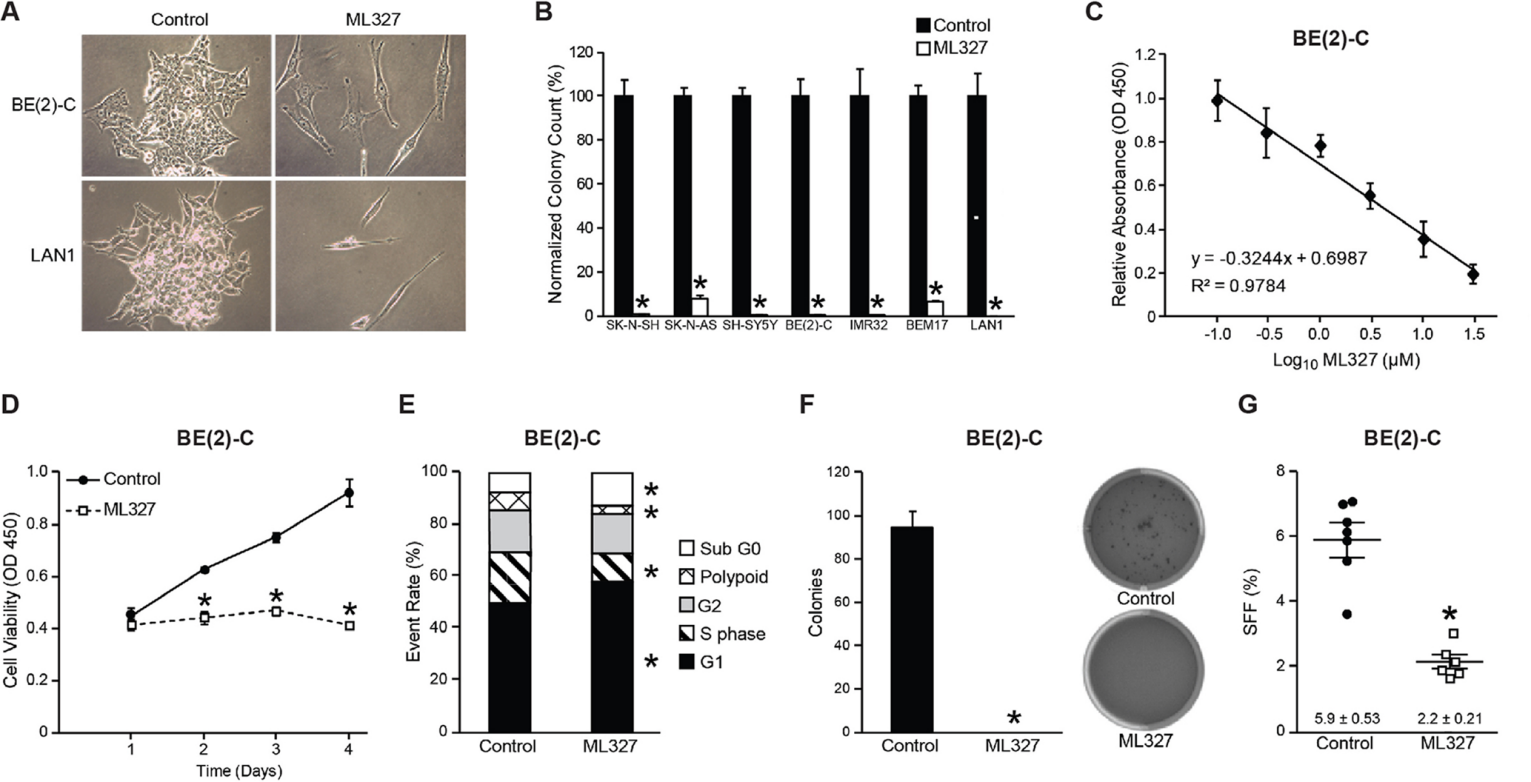
ML327 induces cell death and cell cycle arrest in neuroblastomas (**A**) Light microscopy (20×) demonstrates induction of an elongated phenotype in neuroblastoma cells treated with ML327 (10 µM). (**B**) ML327 (10 µM) inhibits adherent colony formation in *MYCN-*amplified and *MYCN-*single copy neuroblastoma cell lines. (**C**) Representative concentration-dependent cellular viability plot for BE(2)-C cells with an estimated IC50 of 4 µM. (**D**) Time course plot of cellular viability in BE(2)-C cells using CCK-8 colorimetric assay. (**E**) Cell cycle analysis with propidium iodide demonstrates enhanced G1 cell cycle arrest and induction of subG0 phase at 48 h following treatment. (**F**, **G**) Anchorage-independent growth and sphere forming frequency (SFF) is blocked in BE(2)-C cells by treatment with ML327 (10 µM).

Based on our prior observations in carcinoma cell lines, we first sought to determine the effects of ML327 on neuroblastoma growth *in vitro.* For our initial characterization, we performed an adherent colony forming assay on seven neuroblastoma cell lines wherein cells were seeded at a clonal density and treated with either ML327 (10 μM) or vehicle for a period of 7–10d (Figure 1A, 1B). Treatment with ML327 induced an elongated morphology in neuroblastoma cells. Representative images of BE(2)-C and LAN1 neuroblastoma cells are shown (Figure 1A). These phenotypic changes were associated with marked inhibition (at least 14-fold) in adherent colony formation in all of the tested neuroblastoma cell lines (Figure 1B). We then utilized our tetrazolium-based assay to conduct a concentration response analysis of ML327 in BE(2)-C, LAN1, IMR32, and SH-SY5Y cell lines to estimate the IC50 value for ML327 after 72 h treatment (Figure 1C, Supplementary Figure 1A–1C). The calculated IC50s for BE(2)-C, LAN1, IMR32, and SH-SY5Y were 4, 6, 10, and 5 μM, respectively (Figure 1C, Supplementary Figure 1A–1C). Thus, ML327 inhibits neuroblastoma growth in the low micromolar range (< 10 μM) *in vitro*. To demonstrate the timing of growth inhibition, we treated neuroblastoma cell lines with ML327 (10 μM) and measured cellular viability over a 4d time course (Figure 1D, Supplementary Figure 1D–1F). Significant growth inhibition was first noted by Day 2 in BE(2)-C and SH-SY5Y cells and by Day 3 in LAN1 and IMR32 cells.

We performed cell cycle analysis using flow cytometry with propidium iodide to determine how ML327 is altering neuroblastoma growth. BE(2)-C cells treated with ML327 demonstrated G1 cell cycle arrest with a concordant decrease in S phase population, and a significant increase in the sub G0 population (Figure 1E). Enhancement of the subG0 population represents cells that are undergoing DNA fragmentation and cell death. Taken together, these findings demonstrate that ML327 impairs neuroblastoma growth *via* induction of G1 cell cycle arrest and cell death.

We further validated the effects of ML327 on neuroblastoma growth *in vitro* by quantifying anchorage-independent growth and sphere-forming frequency. Anchorage-independent growth is generally regarded as a hallmark of transformation, while the sphere-forming population of neuroblastomas has an enriched tumor-initiating capacity [15, 16]. *I-type* BE(2)-C cells are high sphere and colony-forming neuroblastoma cell lines [17, 18]. Concurrent treatment with ML327 completely abolished the ability of BE(2)-C cells to develop soft agar colonies (Figure 1F; 0 vs. 95 colonies; *p* < 0.0001). Furthermore, we estimated the sphere-forming frequency of BE(2)-C cells in the presence of ML327 and demonstrated a marked inhibition in sphere formation (Figure 1G; 2.2 vs. 5.9%; *p* < 0.0001) in the presence of ML327(10 μM). These findings suggest that ML327 represses the transformation potential and may block the tumor-initiating cell population of neuroblastomas.

### ML327 induces a neuroepithelial-like differentiation signature in neuroblastoma

**Figure 2:**
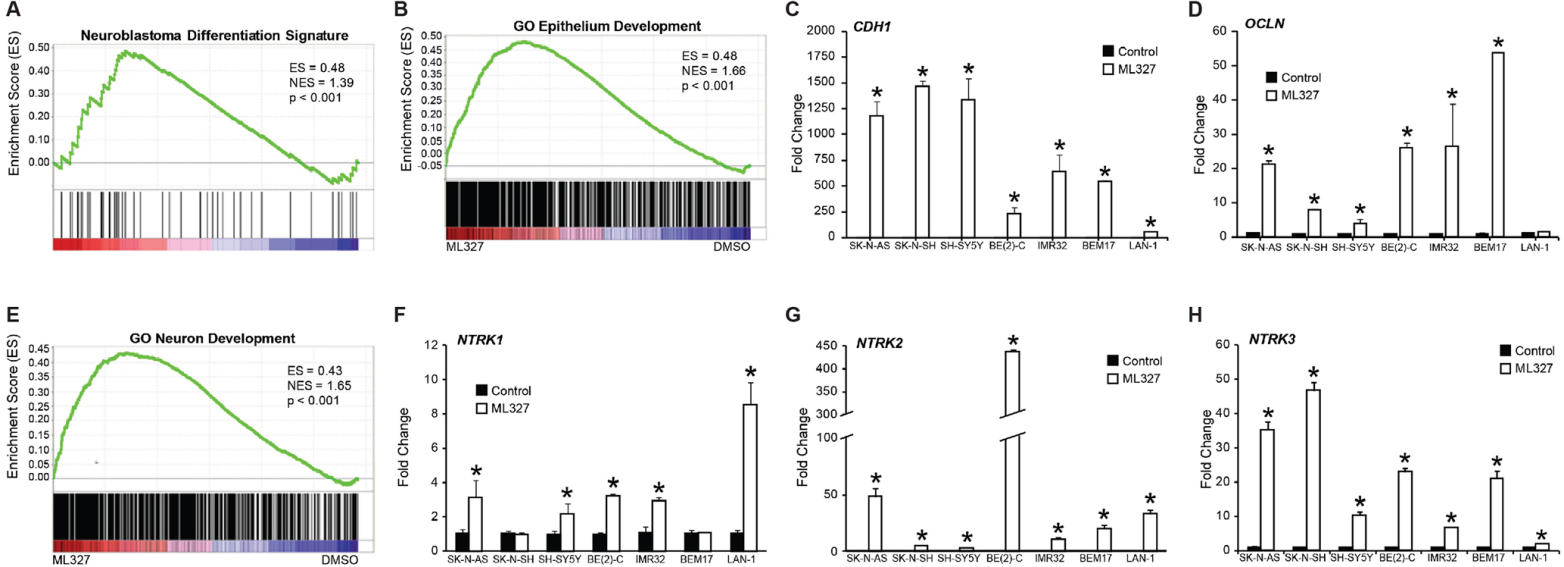
Effects of ML327 on neuroepithelial differentiation RNA sequencing analysis of BE(2)-C cells treated with ML327 and vehicle for 7d. (**A**) GSEA for the neuroblastoma differentiation signature gene set. ES, enrichment score; NES, normalized enrichment score. (**B**) GSEA for gene ontology gene set for epithelial development. (**C**, **D**) RT-PCR survey of neuroblastoma cell lines for the expression of epithelial hallmarks, *CDH1 and OCLN.* (**E**) GSEA for gene ontology gene set for neuronal development. (**F**, **G**, **H**) RT-PCR survey of neuroblastoma cell lines for the expression of neuronal markers, *NTRK1*, *NTRK2*, and *NTRK3.*

Based upon the morphologic, behavioral, and cell cycle arrest features induced by ML327, we postulated that ML327 enhanced neuroblastoma differentiation. We completed RNA sequencing utilizing RNA isolated from BE(2)-C cells treated with ML327 or vehicle for 7d (Supplementary Table 1). For our initial characterization, we utilized the neuroblastoma differentiation signature described by Frumm et al. [19]. Gene set enrichment analysis demonstrated enrichment of the neuroblastoma differentiation signature with ML327 treatment (Figure 2A; Supplementary Figure 2A). Further, enrichment analysis for gene ontology demonstrated significant enrichment of epithelial (Figure 2B; Supplementary Figure 2B) and neuronal development (Figure 2E; Supplementary Figure 2C), and neuroepithelial cell differentiation (Supplementary Figure 2F, 2G). Expression of E-cadherin (*CDH1*) and the formation of Occludin-containing (*OCLN*) tight junctions are two hallmarks of epithelial differentiation. ML327 induced the expression of *CDH1* in all seven of the neuroblastoma cell lines with a 50 to 1,400-fold induction of *CDH1* mRNA expression (Figure 2C). More modest increases in *OCLN* expression were also observed*,* ranging from 1.5 to 50-fold induction (Figure 2D). Other notable epithelial markers enriched by ML327 in our RNA sequencing analysis include: *CD109, Gli3, CDH3* (Supplementary Figure 2B). The TRK family of neurotrophin receptor tyrosine kinases are neuronal hallmarks that play critical roles in neuroblastoma survival and differentiation. Specifically, TrkA (*NTRK1)* and C (*NTRK3*) expression are associated with favorable outcomes in neuroblastoma, while TrkB (*NTRK2*) is associated with biologically aggressive disease [20]. We surveyed our seven neuroblastoma cell lines for *NTRK1,2,3* expression and demonstrated enhanced mRNA levels for *NTRK2 and NTRK3* in all tested cell lines (Figure 2G, 2H) , as well as enhanced *NTRK1* expression in 5 of 7 experimental cell lines (Figure 2F). Taken together, these findings suggest that ML327 induces a gene signature consistent with neuroblastoma differentiation, featuring characteristics of partial neuroepithelial differentiation.

### ML327 blocked MYC expression in neuroblastomas

**Figure 3:**
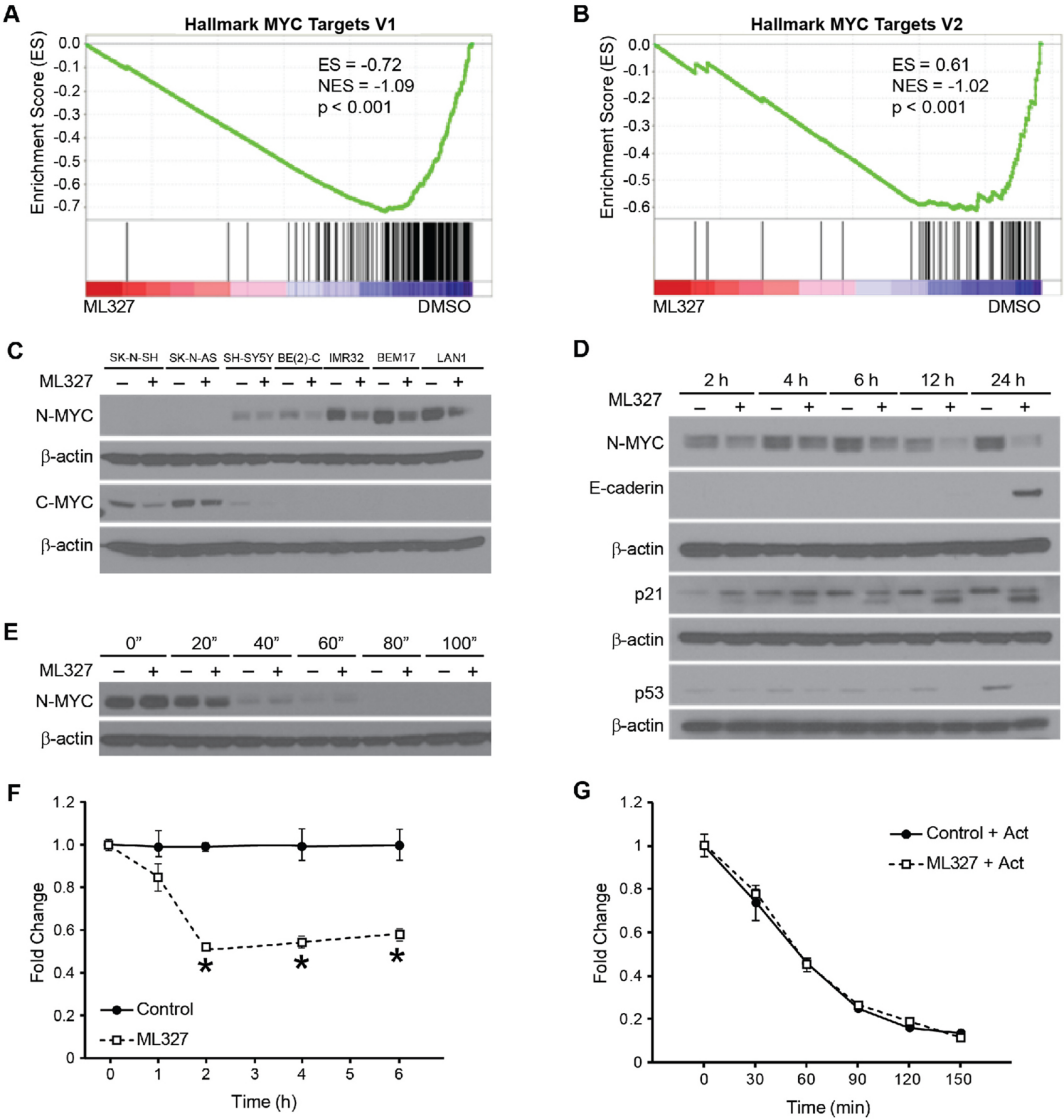
ML327 blocks MYC signaling in neuroblastoma (**A**, **B**) RNA sequencing demonstrates repression of hallmark MYC target gene sets by GSEA (**C**) Western blots demonstrate that ML327 inhibits C-MYC expression and N-MYC protein expression levels in *MYCN*-single and *MYCN-*amplified neuroblastoma cell lines, respectively. β-actin was used for protein loading control. (**D**) Immunoblot demonstrating time course of N-MYC, p21/Cip1, p53, and E-cadherin protein expression changes in the presence and absence of ML327. (**E**) Co-treatment with cycloheximide demonstrates that ML327 fails to alter protein half-life of MYC. (**F**) RT-PCR demonstrates early changes in *MYCN* mRNA levels in the presence of ML327. (**G**) Co-treatment with actinomycin D demonstrates that ML327 fails to alter mRNA half-life.

To further elucidate a potential mechanism by which ML327 may be exerting its anti-tumorigenic effects in neuroblastoma, we performed gene set enrichment analysis utilizing the hallmark gene sets provided through the Broad Institute. Of interest, we determined that two hallmark MYC target gene sets were negatively associated with ML327 treatment (Figure 3A, 3B; Supplementary Figure 2D, 2E). These findings were supported by a five-fold reduction repression of *MYCN* transcription levels in our BE(2)-C cells by RNA sequencing (Supplementary Table 1). This finding led us to hypothesize that ML327 may be repressing MYC signaling in our neuroblastoma cell lines.

Thirty percent of neuroblastomas have *MYCN-*amplification featuring elevated N-MYC expression, while the C-MYC is predominantly expressed in *MYCN*-single copy neuroblastomas. We first validated that ML327 represses MYC signaling by demonstrating repression of N-MYC protein expression in the treatment of 4 *MYCN-*amplified neuroblastoma cell lines and C-MYC repression in 3 *MYCN*-single copy cell lines (Figure 3C). Thus, ML327 blocked the expression of MYC family of oncogenic transcription factors in all tested neuroblastoma cell lines.

We further characterized the timing and mechanism of N-MYC repression using our *MYCN*-amplified BE(2)-C cell line. Immunoblotting time course demonstrated early repression of N-MYC expression within 2 h of treatment with ML327 (10 µM; Figure 3D). P21/Cip1 expression was also induced by 2 h. Changes in N-MYC and P21/Cip1 expression temporally preceeded the reexpression of E-cadherin. Intriguingly, p53 levels were suppressed by treatment with ML327, suggesting that MYC repression and p21 induction occurs *via* a p53-independent mechanism (Figure 3D). To further clarify the mechanism of N-MYC repression, we completed cycloheximide chase experiments demonstrating no significant changes in protein half-life between ML327 treatment and vehicle control treated cells (36 vs. 31 min; Figure 3E). Also, *MYCN* mRNA expression levels were significantly decreased two-fold within 2 h of treatment suggesting that the decrease in N-MYC levels after ML327 treatment was due to downregulation of *MYCN* mRNA expression (Figure 3F). No changes in mRNA stability were noted with co-treatment with actinomycin D (Figure 3G). As such, ML327 treatment appears to result in decreased *MYCN* transcription.

Bromodomain inhibitors, such as JQ1, have demonstrated considerable preclinical promise for the treatment of MYC-driven malignancies, including neuroblastomas [21, 22]. We sought to determine whether JQ1 was able to recapitulate the epithelial features observed with ML327 treatment. Notably, JQ1 failed to induce E-cadherin protein expression (Supplementary Figure 3A). We also sought to determine whether RNA interference of MYC was sufficient for the induction of epithelial differentiation in neuroblastoma. MYC silencing alone failed to induce E-cadherin re-expression and MYC silencing failed to augment E-cadherin expression in the presence of ML327 (Supplementary Figure 3B). Taken together, these findings suggest that ML327 both represses the MYC signaling cascade and independently modulates the expression of critical epithelial markers, such as E-cadherin.

### ML327 pretreatment blocked the proliferative potential and tumor-initiating capacity of neuroblastomas

**Figure 4:**
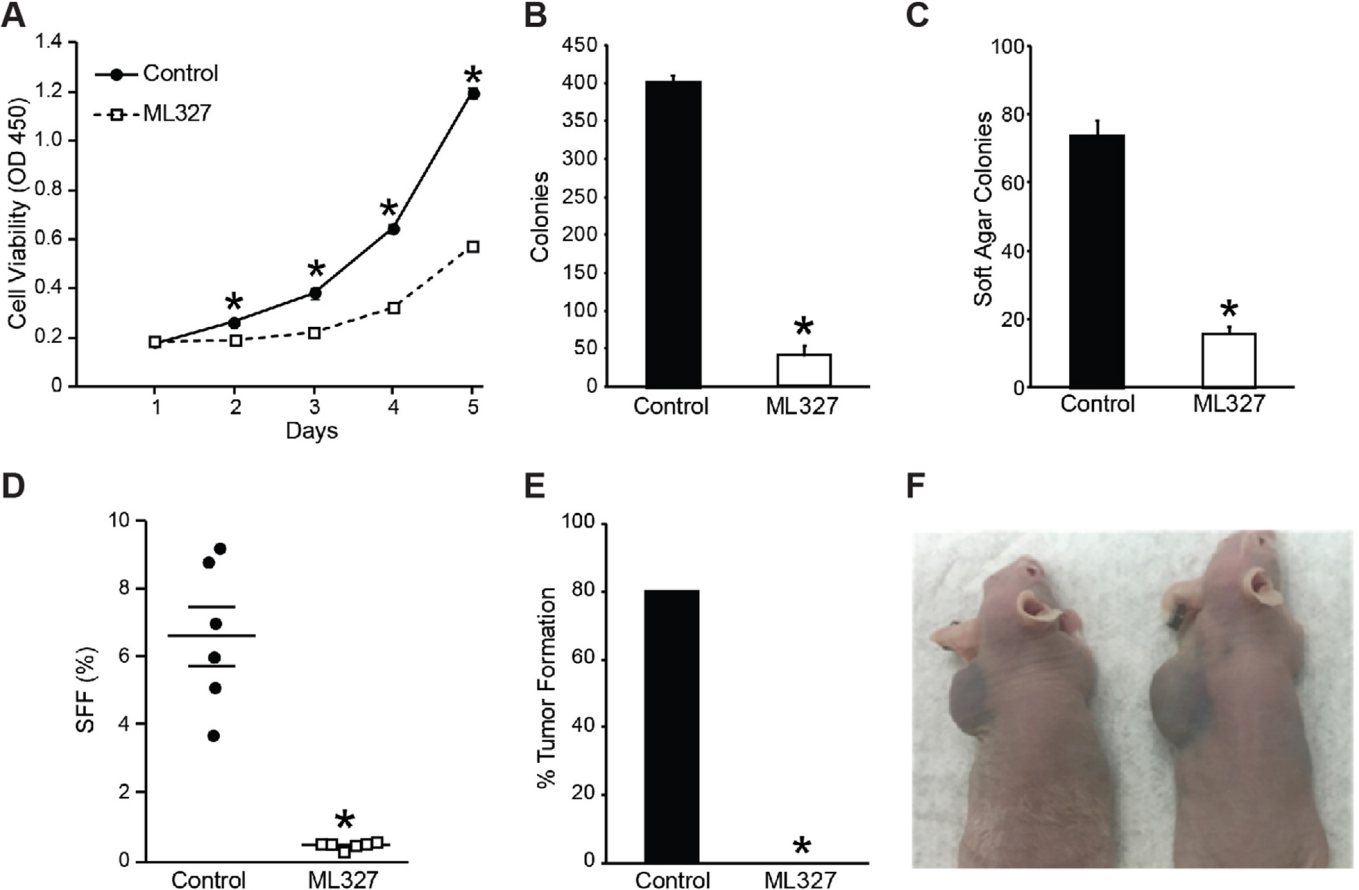
Pretreatment with ML327 blocks neuroblastoma proliferative potential and tumor-initiating capacity Pretreating BE(2)-C cells with ML327 (10 µM) decreased proliferative potential as measured by (**A**) cell viability with CCK-8 assay, (**B**) adherent clonogenesis, (**C**) soft-agar colony formation, and (**D**) sphere-forming frequency. (**E**) Pretreatment (7d) of BE(2)-C cells with ML327 limits their tumor-initiating capacity in a subcutaneous xenograft model. (**F**) BE(2)-C cells pretreated with vehicle were injected into the left subscapular region, while ML327-pretreated cells with injected on the right side. Representative images were obtained two weeks following injection.

Given that ML327 induces transcriptional, morphologic, and cell cycle arrest features consistent with differentiation, we sought to determine whether pretreatment with ML327 alters the proliferative potential and tumor-initiating capacity of neuroblastoma cells even after withdrawal of compound. BE(2)-C cells were pretreated for 1 week with ML327 (10 µM) or vehicle and subsequently plated to quantify proliferation, adherent colony formation, soft agar colony formation, and neurosphere formation. Intriguingly, ML327-pretreated cells demonstrated reduced proliferative potential in both tetrazolium-based (*p* < 0.0001) and adherent 2D colony formation (41 vs. 400; *p* < 0.0001; Figure 4A, 4B) demonstrating persistent defects in proliferation even after removal of ML327. Similarly, ML327-pretreated BE(2)-C cells grew significantly fewer soft agar colonies (Figure 4C; 74 vs. 16; *p* < 0.0001 ) and neurospheres (Figure 4D; 6.6 vs. 0.4%; *p* < 0.0001 ), as compared with vehicle-pretreated cells. As such, pretreatment with ML327 decreases the proliferative potential, anchorage-independent growth, and tumor-initiating cell population of neuroblastomas.

Given these persistent *in vitro* findings, we next evaluated whether pretreatment with ML327 could alter the ability of BE(2)-C cells to develop subcutaneous xenografts. After 7d of pretreatment, identical numbers (1 × 10^6^) of vehicle pre-treated cells were injected into the left flank, and ML327-pretreated cells were injected into the right flank. After two weeks, 80% (8 out of 10) of vehicle pre-treated cells had developed tumor on the left side, while no tumors (0 out of 10) developed on the right side where ML327-pretreated cells were implanted (Figure 4E, 4F). Together, these findings are consistent with ML327 inducing differentiation in neuroblastomas, as evidenced by persistent defects in proliferative potential and tumor-initiating capacity.

### ML327 blocked growth of established neuroblastoma xenografts

**Figure 5:**
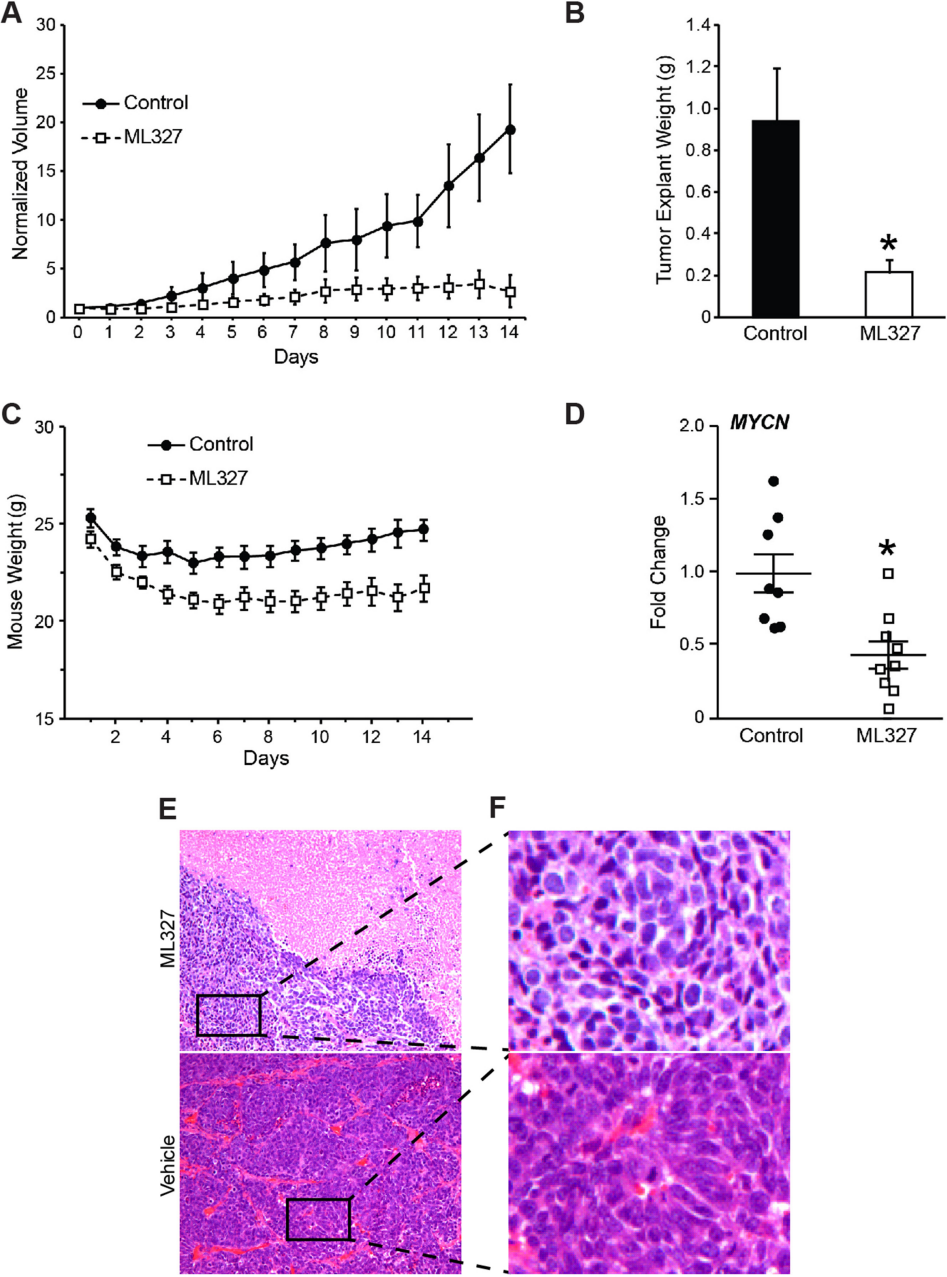
Growth inhibition of neuroblastoma xenografts by ML327 BE(2)-C subcutaneous xenografts were established and randomized to receive intraperitioneal injection with ML327 (50 mg/kg b.i.d.; *N* = 9) or vehicle control (*N* = 8). (**A**) Tumor volumes were measured daily and (**B**) explant weights were obtained at time of sacrifice following 2 weeks of treatment. (**C**) Daily weight measurements demonstrated a 12% weight loss in ML327-treated mice. (**D**) *MYCN* mRNA levels were measured by RT-PCR using RNA isolated from ML327 and control tumors. (**E**) Representative H&E sections were obtained from ML327 and vehicle-treated tumors and demonstrated large areas of necrosis within ML327-treated tumors (10× magnification). (**F**) Residual, viable neuroblastoma cells in ML327-treated remained poorly differentiated (40× magnification).

Given the established role of N-MYC in neuroblastoma tumorigenesis, we sought to characterize the preclinical efficacy of ML327 in established neuroblastoma xenografts. Athymic male mice were injected with 2 × 10^6^ BE(2)-C cells into the subcutaneous flank and allowed to grow into established tumors of 75–100 mm^3^. Mice developing tumors were then randomized to receive either ML327 (50 mg/kg) or vehicle by intraperitoneal twice daily injections for two weeks. Tumor volumes were measured daily. ML327 treatment significantly reduced tumor volume by three-fold over the two-week treatment period (Figure 5A; *p* = 0.02). Similarly, tumor explant weights were approximately three-fold smaller in the ML327-treated mice (Figure 5B; *p* = 0.01).

ML327-treated mice demonstrated normal activity while receiving treatment with ML327. Interestingly, mice treated with ML327 lost 12% more body weight than vehicle treated mice (Figure 5C). The majority of the weight loss occurred in the first four days of treatment, after which their weights stabilized, demonstrating potential side effects that may be associated with ML327 treatment (Figure 5C). We collected blood from four ML327-treated and three vehicle control-treated mice to perform laboratory analysis to discern evidence of toxicity. No significant differences in hematologic, immunologic, liver function, or kidney function were appreciable.

We isolated RNA from ML327 and vehicle-treated tumors to determine whether ML327 had comparable effects on *MYCN* and *CDH1* expression to those observed *in vitro*. Importantly, ML327 treatment results in a two-fold decrease in *MYCN* expression, confirming that ML327 inhibits xenograft *MYCN* expression (Figure 5D; *p* = 0.0035). Intriguingly, *CDH1* levels were not detectable from RNA isolated from any of the xenografts that received two weeks of treatment. Histologic analysis of tumor samples was performed by a blinded, pediatric pathologist (H.C.) who noted large pools of coagulative necrosis within the ML327-treated tumors (∼30% vs. < 10%; Figure 5E). The remaining viable neuroblastoma tissue was poorly differentiated (Figure 5F). Together, these findings suggest that ML327 suppresses *MYCN* levels within neuroblastoma xenografts and induces cell death within xenografts.

We hypothesized that the pools of necrotic cells within the ML327-treated tumors may have expressed *CDH1* earlier in the treatment course. As such, we completed a separate, pilot study to evaluate whether *CDH1* expression was induced with a shorter duration (5 doses) of ML327 treatment (50 mg/kg b.i.d). With this shorter treatment course, *CDH1* expression was detectable and the mRNA levels were robustly induced compared to vehicle-treated tumors (Supplementary Figure 4). This pilot study validates that ML327 is capable of inducing *CDH1* expression within neuroblastoma xenografts and demonstrates conservation of the gene signature changes observed *in vitro.*

## DISCUSSION

Neuroblastomas demonstrate considerable clinical and biologic heterogeneity with select tumors spontaneously regressing while others progress despite our most aggressive interventions [2, 3, 20]. Herein, we hypothesized that ML327, an MET induction agent identified in epithelial carcinomas, would elicit anti-tumorigenic effects in neural crest-derived neuroblastomas. Intriguingly, ML327 elicited striking changes in cell viability not previously observed in epithelial-derived cancers [14]. We attribute these alterations in cell viability to induction of cell death and features of neuroblastoma differentiation *in vitro*. Specifically, ML327 successfully induces cell cycle arrest, morphologic changes, and gene expression signatures consistent with neuroepithelial differentiation (Figures 1, 2; Supplementary Figure 2). We also demonstrate significant induction of the subG0 population by propidium iodide-based cell cycle analysis consistent with the onset of DNA fragmentation and cell death. Immunoblotting for proteolysis- activated mediators of apoptosis (PARP and Caspase 3) demonstrated minimal induction of PARP cleavage and no Caspase 3 cleavage, suggesting that cell death may be occurring by necrosis (results not shown). Overall, these findings demonstrate the capacity of ML327 to elicit cell death and features of neuroepithelial differentiation within neuroblastoma cell lines *in vitro*. Intriguingly, we were able to block established *MYCN*-amplified xenograft growth with intraperitoneal ML327 administration over a two week period (Figure 5A). The most striking feature observed on histologic evaluation of ML327-treated xenografts was the induction of large pools of coagulative necrosis with remaining, viable tumor being poorly differentiated (Figure 5E, 5F). We were able to demonstrate suppression of *MYCN* within these ML327-treated xenografts, however *CDH1* levels were undetectable by RT-PCR. To remedy the discrepant *CDH1* changes observed *in vitro* and *in vivo*, we conducted a pilot study and demonstrated that *CDH1* was significantly enriched with a shorter period ML327 treatment (Supplementary Figure 4). Our working hypothesis is that there is a heterogenous uptake of ML327 within the xenografts and those neuroblastoma cells that express E-cadherin in the presence of ML327 subsequently succumb to cellular necrosis. This possibility remains hypothetical and is a focus of on-going investigations.

C-MYC and N-MYC are the two predominant pro-tumorigenic members of the MYC family driving cell cycle progression, angiogenesis, genomic instability, and metastasis [5, 23]. These features highlight the therapeutic potential of targeting the MYC family oncogenes in the treatment of both pediatric and adult malignancies. Directly targeting the MYC protein family has proven to be a vexing effort [23, 24]. Recent advances have been made in targeting the transcription of MYC through bromodomain inhibition or promoting protein degradation utilizing aurora kinase inhibitors [21]. Both of these families of compounds have demonstrated preclinical efficacy with inhibition of neuroblastoma cell growth, but early reports of resistance have emerged [25–28]. In this study, we have identified a novel isoxazole-based compound that similarly destabilizes the MYC signaling cascade. Notably, we have demonstrated both blockade of N-MYC and C-MYC expression, respectively within the *MYCN*-amplified and *MYCN-*single copy cell lines. ML327 appears to transcriptionally regulate MYC mRNA levels. These findings are associated with the induction of cell cycle arrest and blockade of anchorage-independent growth and neurosphere formation *in vitro.* Furthermore, we have demonstrated that ML327 is capable of blocking established xenograft growth and changes in *MYCN* mRNA levels *in vivo*.

A critical deficiency of our experiments with ML327 remains that we have yet to identify a direct intracellular effector of this small molecule. Efforts at labeling ML327 with radioisotopes, biotin, and iodine result in a loss of compound efficacy *in vitro* and identifying its potential binding partner is the focus of ongoing studies. However, the phenotypic responses and gene signature elicited by ML327 treatment have considerable therapeutic potential. In particular, MYC can induce EMT in driving tumor progression, but the reports demonstrate that this occurs post-transcriptionally [29]. In conjunction with our previous work, we have demonstrated that ML327 is a novel small molecule that both represses *MYC* transcription and transcriptionally activates *CDH1* in both colon cancer cells and neuroblastomas [14]. MYC silencing utilizing RNA interference is not sufficient to induce E-cadherin, and bromodomain inhibition fails to initiate reexpression of E-cadherin in BE(2)-C cells despite successful MYC blockade, highlighting the unique properties of ML327 (Supplementary Figure 3).

The transcriptional plasticity of MYC expression has led to early development of resistance to bromodomain inhibitors [25, 27, 28]. Specifically, Wnt-activation has been previously shown to mediate the reactivation of MYC signaling in leukemia cells resistant to bromodomain inhibition [27]. Dual-treatment strategies with ML327 and bromodomain inhibitors offer the potential both for additive MYC blockade and the potential tumor suppressor effects of E-cadherin. Intriguingly, E-cadherin has been shown to bind to and block β-catenin nuclear localization [30], suggesting a potential mechanism by which ML327 may potentiate or re-sensitize cancers to bromodomain inhibition. Such theoretical considerations merit further investigation and highlight the need to identify the precise mechanisms by which ML327 acts.

In conclusion, we have identified the isoxazole-based compound ML327 as a novel inhibitor of the MYC signaling cascade in neuroblastoma. These features inhibit the transformation potential, tumor-initiating capacity, and established xenograft growth of neuroblastomas. MYC signaling has previously been shown to have considerable transcriptional plasticity, and ML327 may have potential efficacy both alone or in conjunction with existing therapeutics. Future identification of the binding partner of ML327 will both inform future drug discovery efforts and enhance our understanding of MYC regulation.

## MATERIALS AND METHODS

### Antibodies and reagents

Primary antibodies were obtained for N-MYC from Santa Cruz (Dallas, TX). E-cadherin antibodies were from Cell Signaling Technology (Danvers, MA), and p21/Cip1 and PARP primary antibodies were obtained from Abcam (Cambridge, MA). All other reagents were obtained from Sigma (St. Louis, MO).

### Chemical synthesis

ML327 was synthesized as previously described through the Vanderbilt Institute of Chemical Biology [14]. ML327 was solubilized in DMSO for *in vitro* trials and 70% polyethylene glycol for *in vivo* experiments.

### Cell culture

LAN1 was a gift from Dr. Robert C. Seeger (University of Southern California, Los Angeles, CA). All other neuroblastoma cell lines (BE(2)-C, BEM17, IMR32, SK-N-SH, SK-N-AS, and SH-SY5Y) were purchased from the American Type Culture Collection (ATCC, Manassas, VA). Cells were maintained in RPMI 1640 with 10% FBS at 37°C in a humidified atmosphere consisting of 5% CO_2_ and 95% air.

### Clonogenesis assay

Cells were plated at clonal density (1,000–5,000 cells/well), permitted to attach, and then treated with either ML327 (10 μM) or vehicle (control) at 72 h intervals over a 7–10 d period. Colonies were stained with 0.05% crystal violet, photographed, and counted in triplicate.

### Cell viability assay

Cells were seeded onto 96-well plates at equivalent density (3,000–10,000 depending upon cell line), permitted to attach overnight, and treated with either ML327 (10 μM) or vehicle. Daily absorbance measurements (450 nm) using the Cell Counting Kit-8 (CCK-8; Dojindo Molecular Technologies, Rockville, MD) were obtained. For estimation of IC_50_ values, cells were plated at equal density, permitted to attach, and baseline absorbance was obtained using CCK-8. Cells were then treated with varying doses of ML327 (0.1–30 μM) and cell viability was measured 72 h after treatment.

### Soft agar colony formation assay

Cells were trypsinized and resuspended in RPMI medium 1640 containing 0.4% agarose and 5% FBS. BE(2)-C cells were overlaid onto a bottom layer of solidified 0.8% agarose in RPMI medium 1640 containing 5% FBS and incubated for 2 weeks. Colonies were stained with 0.05% crystal violet, photographed, and quantified.

### Sphere formation assay

Neurosphere formation was quantified by performing serial limiting dilution analysis in the presence and absence of ML327 (10 μM). Spheres were reared in neurobasal media supplemented with B27, epidermal growth factor (20 ng/mL), and fibroblast growth factor (20 ng/mL; Invitrogen, Carlsbad, CA). Cells were plated onto an ultra-low attachment 96-well plate at serial dilutions. Each well was scored for presence of sphere formation as previously described [31].

### Cell cycle analysis

Cell cycle distribution was analyzed using flow cytometry. 1 × 10^6^ cells were trypsinized, washed with PBS, and fixed in 70% ethanol. Fixed cells were incubated with RNAse (100 μg/ml), stained with propidium iodide (50 μg/ml), and analyzed on a 3-laser BD LSRII (BD Biosciences, San Jose, CA). Flow Cytometry experiments were performed in the VUMC Flow Cytometry Shared Resource.

### RT-PCR and western blot analyses

Total RNA was isolated and purified using an RNeasy isolation kit with DNAse digestion. cDNA was synthesized using the High-Capacity cDNA Reverse Transcription Kit (Applied Biosystems, Carlsbad, CA). Specific target primers are, *MYCN* (forward 5′-GCTTCTACCCGGACGAAGATG-3′; reverse 5′-CAGCTCGTTCTCAAGCAGCAT -3′), *NTRK1* (forward 5′-TTCCATTTCACTCCTCGGCTCAGT-3′; reverse 5′-ACGTCACGTTCTTCCTGTTGAGGT-3′), *NTRK2* (forward 5′-TCAATGCCAGGCAGGTCTCCTAAA-3′; reverse 5′-TTGGTGCAGAATTCCCAGCAAAGG), *NTRK3* (forward 5′-TGCAGTCCATCAACACTCACCAGA-3′; reverse 5′-TGTAGTGGGTGGGCTTGTTGAAGA-3′), *OCLN* (forward 5′-AGGAACCGAGAGCCAGGT -3′;reverse 5′-GGATGAGCAATGCCCTTTAG-3′), *CDH1* (forward 5′-TTGACGCCGAGAGCTACAC-3′; reverse 5′-GTCGACCGGTGCAATCTT-3′), *GAPDH* forward 5′-GTTCCAATATGATTCCACCC -3′;reverse 5′-TGAGTCCTTCCACGATACC -3′). Amplification was performed for 40 cycles of 30 s at 95°C, 30 s at 55°C, and 40 s at 72°C. GAPDH was used as a control. Western blot analysis was performed using β-actin as a loading control as described [32].

### RNA sequencing and gene set enrichment analysis

BE(2)-C neuroblastoma cells (*n* = 3 per group) were reared in DMSO or ML327 for 7d, and RNA was collected using RNeasy kits with DNAse treatment. Processing of RNA using a TruSeq Stranded mRNA sample prep kit was conducted according to the manufacturer’s instructions (Illumina, San Diego, CA). Approximately 30 million 75 base pair single-end reads were generated, per sample. We mapped the reads to the human genome hg19 using TopHat-2.0.10 [33]. Then, we counted the number of reads that fall into annotated genes by samtools-0.1.19 [34] and HTSeq-0.5.4p5 [35] using the method described by Anders et al. [36]. For gene set enrichment analysis, gene set collections were from the Molecular Signatures Database (http://www.broadinstitute.org/gsea/msigdb/).

### Tumor-initiating capacity

BE(2)-C cells were pretreated for 1 week with ML327 (10 μM) or vehicle (DMSO). Following 7 d of treatment, cells were trypsinized and resuspended in HBSS at 1 × 10^6^ cells/100 μL. ML327 and vehicle-pretreated cells were injected into the subcutaneous tissue lateral to the right and left scapular region, respectively. Mice were monitored twice weekly for the development of tumors over a two-week period.

### Growth of established xenografts

Male athymic nude mice (4–6 weeks old) were maintained as described [37]. All studies were approved by the Institutional Animal Care and Use Committee at Vanderbilt University. BE(2)-C cells xenografts were established as previously described [37]. Briefly, 1 × 10^6^ cells/100 µL of HBSS were injected subcutaneously into flanks using a 26-gauge needle (*n* = 10 per group). Mice were monitored daily for xenograft formation and assessed by measuring the two greatest perpendicular tumor diameter with venier calipers (Mitutoyo, Aurora, IL). Xenograft volumes were estimated using the following formula [(length *x* width^2^)/2]. Once tumors reached 75–100 mm^3^, mice were randomized to receive either 50 mg/kg of ML327 or control vehicle (70% polyethylene glycol) via intraperitoneal injection twice daily for 14d. Weight and tumor volume were recorded daily. After completion of two weeks of treatment, mice were euthanized and tumors were excised, weighed, and RNA was isolated.

### Statistical analysis

For *in vitro* experiments, Student’s *t*-test or one-way ANOVA with Tukey correction were used for two or ≥ 3 group experiments, respectively. Tumor explant weight was compared using Student’s *t*-test. Serial tumor volume measurements were compared using a mixed effects model in STSS using fixed effects of treatment and day with a random effect of mouse. For all experiments, *p <* 0.05 was considered significant.

## SUPPLEMENTARY MATERIALS FIGURES AND TABLE





## Abbreviations

EMT: epithelial-to-mesenchymal transition
MET: mesenchymal-to-epithelial transition
