# Supplementary material for: Isoxazole compound ML327 blocks MYC expression and tumor formation in neuroblastoma
Source: Oncotarget. 2017 Jul 20;8(53):91040–51. doi: 10.18632/oncotarget.19406 (PMC5710904; doi:10.18632/oncotarget.19406)
Supplement: Supplementary file 1 [file oncotarget-08-91040-s001.pdf]

Isoxazole compound ML327 blocks MYC expression and tumor formation in neuroblastoma

SUPPLEMENTARY MATERIALS

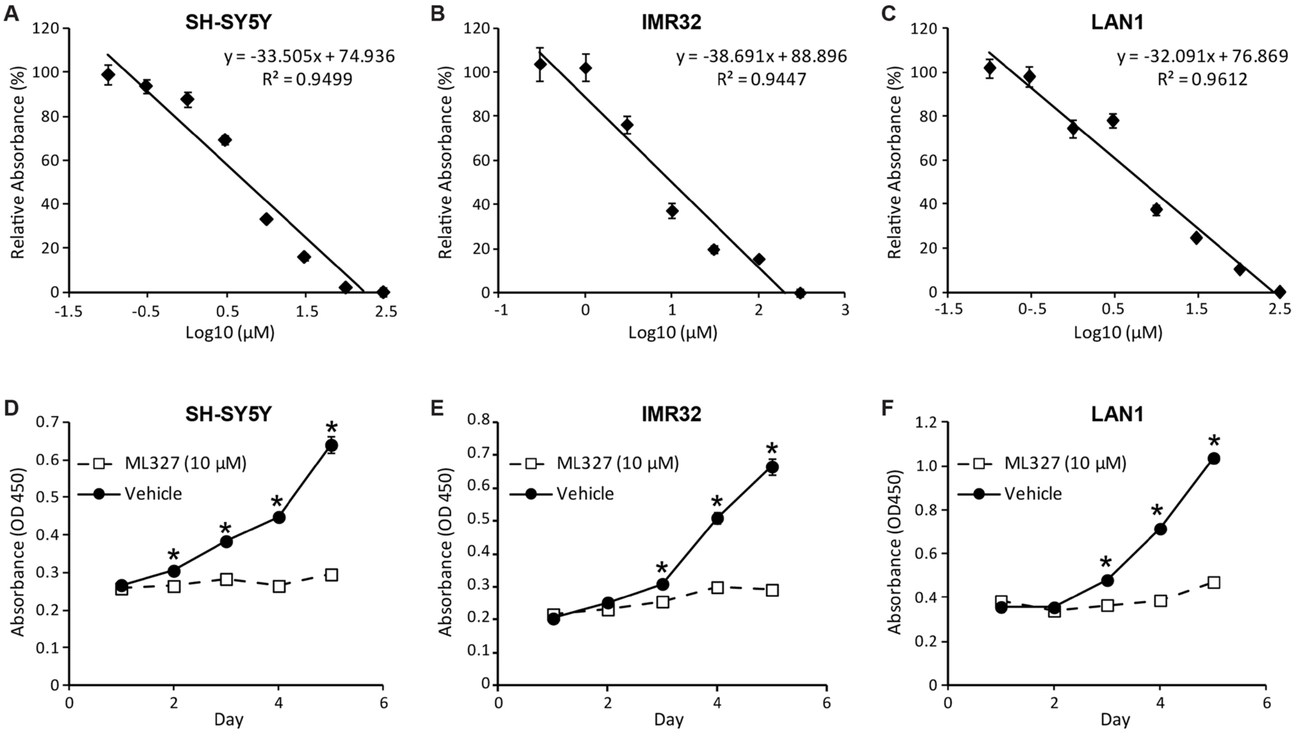

Supplementary Figure 1: ML327 inhibits neuroblastoma growth in MYCN-amplified and MYCN-wildtype cell lines. (**A–C**) Concentration-dependent cellular viability assays were performed to estimate an IC50 for SH-SY5Y (5 µM), IMR32 (10 µM), and LAN1 (6 µM) cells. (**D–F**) Time course plot of cellular viability in SH-SY5Y, IMR32, and LAN1 cells in the presence and absence of ML327 (10 µM) using CCK-8 colorimetric assay.

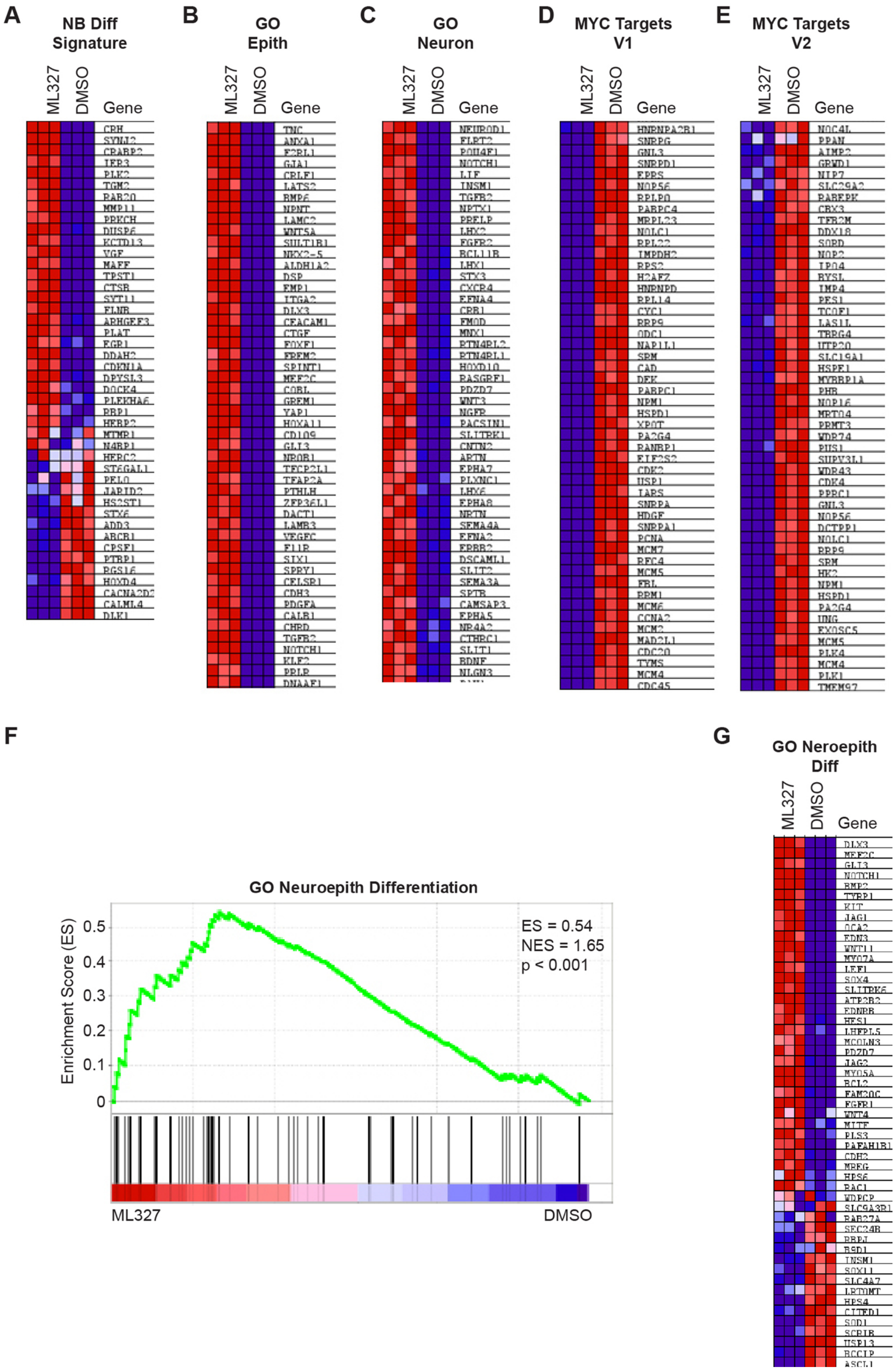

Supplementary Figure 2: RNA sequencing analysis of BE(2)-C cells treated with ML327 (10 µM) and vehicle for 7d. Heat maps of top 50 differentially expressed genes for (**A**) neuroblastoma differentiation signature, (**B**) gene ontology for epithelial development, (**C**) gene ontology for neuronal development, (**D**) hallmark MYC Targets (V1) and (**E**) hallmark MYC Targets (V2). (**F**) GSEA performed using gene ontology for neuroepithelial cell differentiation gene set. (**G**) Heat map for demonstrating differentially expressed genes in the neuroepithelial cell differentiation gene set.

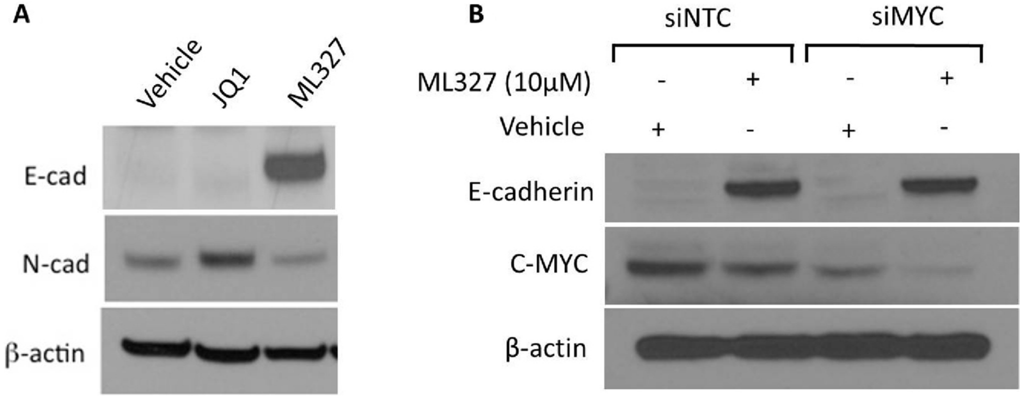

Supplementary Figure 3: (**A**) Immunoblotting demonstrates that JQ1 fails to induce to expression of E-cadherin in BE(2)-C cells. (**B**) Silencing *MYC* expression fails to augment E-cadherin protein expression alone or in combination with ML327 in SK-N-AS cells.

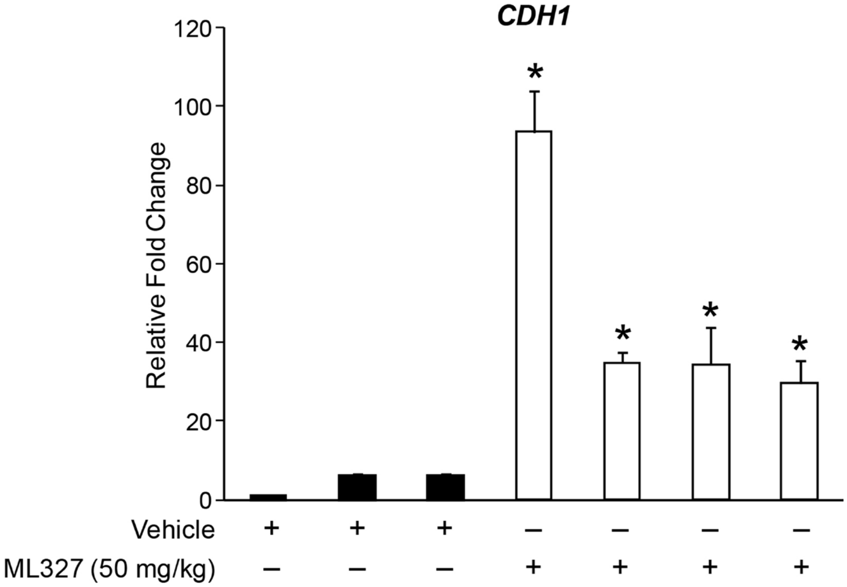

Supplementary Figure 4: ML327 induces CDH1 expression *in vivo*. A pilot study was performed in BE(2)-C xenografts, where RNA was isolated from ML327 (50 mg/kg b.i.d) and vehicle-treated tumors following 5 doses. RT-PCR demonstrates the *CDH1* expression was significantly enhanced in all ML327-treated tumors.

Supplementary Table 1: Differential gene expression list for RNA sequencing performed on BE(2)-C cells treated with vehicle control or ML327 (10 μM) for 7d. See Supplementary_Table_1
